# Long-term dynamics, population structure and connectivity of the helmet jellyfish *Periphylla periphylla* in a Norwegian fjord and adjacent waters

**DOI:** 10.1093/plankt/fbad050

**Published:** 2023-11-29

**Authors:** Nicole Aberle, Charlotte Volpe, Mari-Ann Østensen, Sanna Majaneva

**Affiliations:** Trondhjem Biological Station, Department of Biology Norwegian University of Science and Technology (NTNU), Trondheim 7012, Norway; Institute of Marine Ecosystem and Fishery Science (IMF), Hamburg University, Hamburg 20148, Germany; Trondhjem Biological Station, Department of Biology Norwegian University of Science and Technology (NTNU), Trondheim 7012, Norway; Fisheries and New Biomarine Industry, SINTEF Ocean, Trondheim 7465, Norway; Trondhjem Biological Station, Department of Biology Norwegian University of Science and Technology (NTNU), Trondheim 7012, Norway; Trondhjem Biological Station, Department of Biology Norwegian University of Science and Technology (NTNU), Trondheim 7012, Norway; Ecosystems, Akvaplan Niva, Trondheim 7010, Norway

**Keywords:** connectivity, jellyfish bloom, population genetics, Scyphozoa, zooplankton, Coronatae

## Abstract

Mass occurrences of *Periphylla periphylla* in Norwegian fjords cause major concerns related to potential regime shifts that could affect ecosystem stability. 15 years of trawl data (2006–2015), complemented with comprehensive sampling in different areas and seasons (2018–2021) allowed new insights on the dynamics, structure and connectivity of *P. periphylla* populations within and beyond Trondheimsfjorden. Despite assumed population bursts, no clear trend on *P. periphylla* population size in Trondheimsfjorden were identified. Sampling frequency and population size suggest a local reproduction of *P. periphylla*, especially in the inner part of the fjord where young-of-the-year (YOY) individuals occur. Size variations occurred in relation to sampling month, thus pointing at seasonal patterns in growth and reproduction. No distinct population structure of *P. periphylla* populations within Trondheimsfjorden and over larger spatial scales (> 100 km) along the Norwegian coast was observed. Such poor geographic population structure provides evidence for a strong dispersal of *P. periphylla,* potentially triggered by frequent deep-water renewals of the fjords’ basins that enable a high gene flow. Data on *P. periphylla* long-term dynamics, population structure and connectivity provide valuable information for ecosystem state assessments and enable the advancement of ecosystem management approaches, thus accounting for both stakeholder and ecosystem demands.

## INTRODUCTION

Mass occurrences of jellyfish, both natural and anthropogenic driven, are considered a major challenge that can lead to a restructuring of pelagic ecosystems, clogging of power and desalination plant passages, and negatively impact fisheries, aquaculture and tourism (Baumann and Schernewski, 2012; Doney *et al.,* 2012; Graham *et al.,* 2014; Dong, 2018; Halsband 2018). Jellyfish blooms can especially affect enclosed or semi-enclosed ecosystems such as fjords due to bathymetric or topographic barriers that restrict water exchange, dispersal and distribution patterns with severe impacts on local scales (Mills, 2001). The origin of blooms is often unknown since specimens can be advected from adjacent waters into a given (fjord) ecosystem or originate from local populations, or a combination of these. Reliable estimates on jellyfish population dynamics and stock assessments are scarce thus hindering ecosystem-based management.

The helmet jellyfish *Periphylla periphylla* (Scyphozoa, Coronatae) shows a cosmopolitan distribution with major occurrence in the meso- and bathypelagic zones of the oceans (Arai, 1997; Morita *et al.,* 2017; GBIF.org, 2022). It is considered a strong competitor for fish due to its high growth rates, long life spans and high reproductive success (Jarms *et al.,* 1999; Bamstedt *et al.,* 2020). In contrast to most other metagenetic scyphozoans, *P. periphylla* has a holoplanktonic life cycle with direct development where the planula, ephyra and polyp stages are missing (Jarms *et al.,* 1999, 2002). The development of this species is divided into 14 stages (oldest stages 14 A-D) reaching maturity in development stage 14D with a coronal diameter (CD) > 7.5 cm and an age of ca. 3–4 years (Jarms *et al.,* 2002). Despite its principal distribution in the deep oceans, diel vertical migration in *P. periphylla* is common and occurrences even close to the surface have been reported (Youngbluth and Bamstedt, 2001; Dupont *et al.,* 2009; Geoffroy *et al.,* 2018). From the early 1990 onwards, *P. periphylla* has been frequently reported in deep Norwegian fjords such as Lurefjorden, Sognefjorden, Halsafjorden, and Trondheimsfjorden with established, long-lasting aggregations. In these locations, mass occurrences of several orders of magnitudes higher than in the open ocean were documented (Fossa, 1992; Dalpadado *et al.,* 1998; Jarms *et al.,* 2002; Sornes *et al.,* 2007; Tiller *et al.,* 2017). Despite jellyfish blooms can vary both on a spatial and temporal scale, records of such year-around aggregation of jellyfish are not often reported. As these mass occurrences have been reported with northward shifts in distribution, *P. periphylla* has sometimes been reported as invasive species in the fjords (Tiller *et al.,* 2015; Tiller *et al.,* 2017). In Trondheimsfjorden, *P. periphylla* occurrence can be dated almost 100 years back, as specimens are documented in the collection of the NTNU University Museum, Trondheim, Norway (Bakken *et al.,* 2023). Similar to other jellyfish bloom phenomena, considered to be a consequence of anthropogenic stressors i.e. climate change, overfishing (Attrill *et al.,* 2007; Condon *et al.,* 2012; Duarte *et al.,* 2013), the observed northward shift of *P. periphylla* occurrence has been discussed in the light of global change (Tiller *et al.,* 2017; Geoffroy *et al.,* 2018). Whether observations of *P. periphylla* in areas further north reflect in fact a northward shift and, therefore, invasion of new areas or whether this is rather related to increased ecosystem surveillance and sampling frequency remains unknown.

Due to its potential threat to fjord ecosystems and fisheries, several previous studies have analysed life-history dynamics, reproductive success, behavior and impacts of *P. periphylla* populations on artisanal fisheries in Norwegian fjords (Jarms *et al.,* 2002; Kaartvedt *et al.,* 2015; Bozman *et al.,* 2017; Tiller *et al.,* 2017; Bamstedt *et al.,* 2020). However, large uncertainties remain regarding its population dynamics, intra- and interannual variation, population structure and connectivity. To date, the main driving forces shaping *P. periphylla* populations (e.g. abiotic conditions, anthropogenic drivers) remain elusive.

Usually, low advection is considered as the main factor that causes intense blooms of *P. periphylla* (Sornes *et al.,* 2007), leading to local reproduction and populations in different fjords. Thus, genetically different populations of the same species, even with small geographical distance, could exhibit differences in bloom timing and magnitude (Dawson *et al.,* 2015). So far, local reproduction has been reported from Lurefjorden at the southern Norwegian coast from 1993 onwards. Here, small individuals (<1 cm) have been classified as the newest cohort and large specimens as the old cohorts (Jarms *et al.,* 1999). In addition, eggs, and developmental stages of *P. periphylla* are reported from Lurefjorden from mid-1990 with presence of larvae throughout the year and eggs from summer to fall (Jarms *et al.,* 1999). In Trondheimsfjorden, different size classes of *P. periphylla* medusae have been reported from all three fjord basins (Solheim, 2012; Jøssang, 2015). However, these studies point at a high share of both very small and very large individuals specifically in the inner fjord (Beistadfjorden, Verrasundet and Verrabotten), thus suggesting that the “mother” population of *P. periphylla* has established in inner Trondheimsfjorden and that major recruitment events happen there.

## LOCAL REPRODUCTION AND CONNECTIVITY OF THE BLOOMS

By studying long-term dynamics and intra- and interannual variation of *P. periphylla* population size in Trondheimsfjorden over the past 15 years, insights on the population dynamics and potential trends in bloom frequency and intensity could be revealed. In addition, results on population genetics and size–weight relationships of *P. periphylla* can provide information on population structure and connectivity as well as life-history dynamics in Trondheimsfjorden and adjacent Norwegian waters.

The following hypotheses were addressed:

1. H1: *P. periphylla* population size has increased over the last 15 years.
2. H2: Local reproduction of *P. periphylla* is promoted in the inner part of Trondheimsfjorden where populations accumulate.H3:
3. H3: No clear population structure between *P. periphylla* from the inner part of Trondheimsfjorden compared with mid−/outer fjord areas and adjacent waters can be detected.

## MATERIALS AND METHODS

### Study area

**Fig. 1:**
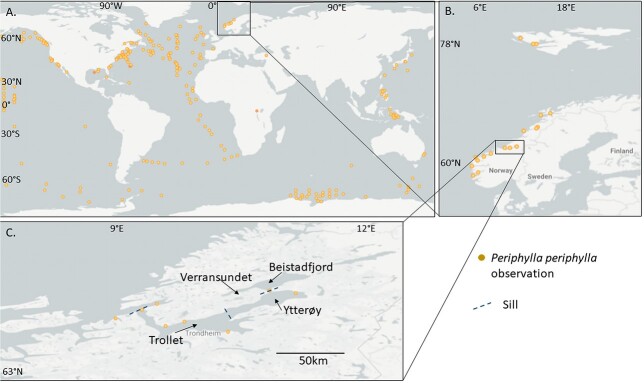
Location with *P. periphylla* observations. (**A**) Global observation based on GBIF database (accessed 1 December 2021) and (**B**) observations in Norway and Svalbard (accessed 1 December 2021) as well as (**C**) in Trondheimsfjorden. Sampling locations in the Outer, Middle and Inner fjord regions are depicted in (**C**) together with the locations of the three sills in Trondheimsfjorden (dashed lines).

Trondheimsfjorden is Norway’s third-longest fjord (total length: 126 km), situated at 63° north on the Norwegian west coast with a maximum depth of 617 m. It is characterised by three main sills that define the different areas of Trondheimsfjorden (Fig. 1): Agdenes sill (separating the fjord from the outer sea), Tautra sill and Skarnsund sill (dividing the fjord into three basins). The basins are Ytterfjorden (Outer fjord), Midtfjorden (Middle fjord) and Beistadfjorden (Inner fjord) (Bakken, 2000). Mixing and transport of water masses in Trondheimsfjorden is affected by wind, river run-off, tidal energy and inflow from the North Atlantic current and the Norwegian Coastal Current (Jacobson, 1983). As the sills in Trondheimsfjorden are quite deep (ranging from 90 to 195 m water depths), an exchange of bottom water masses usually happens twice a year from the North Atlantic via the Norwegian coastal current (Jacobson, 1983). First, from February to May to June, there is an inflow of high salinity Atlantic deep water that produces a new layer of bottom water in the fjord, driving the old water out of the fjord. Secondly, in the late summer period, there is an inflow of 32–34 saline Norwegian intermediate coastal water that mixes into the bottom water and slowly exchanges the old water (Jacobson, 1983). Rivers have a large effect on surface water mixing in Trondheimsfjorden when considering the multiple river sources.

### Long-term *P. periphylla* sampling campaign

A series of research cruises with RV *Gunnerus* were conducted within the framework of several national projects between 2006 and 2015 (LTS, iKyst, Janus) as well as the EU-project GoJelly (2018–2021) where the helmet jellyfish *P. periphylla* was sampled for 15 years. From 2006–2021, bottom trawling using a shrimp trawl (inner lining of fine mesh net, stretched 36 mm mesh size) was conducted sporadically in the inner, mid, and outer part of Trondheimsfjorden at several fixed stations (see Table I). In Midfjorden and Ytterfjorden, trawling took place at the stations Stjørnfjorden, Trollet/Røberg, Tautra and Ytterøya, while the stations in the inner fjord were Beistadfjorden, Verrasundet and Verrabotten. The depth of bottom trawling varied depending on stations between a minimum depth of 50 m at Verrabotten and a maximum depth of 507 m in Trollet. Trawling activities took place throughout the year with major activities in the period from spring to autumn. Specimens of *P. periphylla* were quantified using volumetric measures (L) as an estimate for jellyfish biomass caught during each trawling event. For estimates on the changes in jellyfish biomass over time, the “catch-per-unit effort” (CPUE) for each trawling event was calculated. This was based on the volume of *P. periphylla* (L) caught during a specific bottom trawling per unit of time (min).

**Table I:** Regions, specific locations, geographical coordinates, trawl dates and durations (min.) from bottom trawl deployments in Trondheimsfjorden in the period 2006–2021

Region	Specific location	Trawl date	Geographic coordinates		Trawl duration (min.)	Total *P. periphylla* volume (L)	Total *P. periphylla* abundance (*n*)
Outer fjord	Stjørnfjorden	29 August 2018	N63°43.850’	E09°56.150’	15	7.5	17
		21 September 2020	N63°43.850’	E09°56.150’	10	17.7	40
	Trollet	29 August 2019	N63°29.939’	E10°13.288’	10	4	9
		23 September 2019	N63°29.939’	E10°13.288’	10	18.1	41
		27 August 2020	N63°29.939’	E10°13.288’	5	215.5	487
	Røberg	12 April 2007	N63°28.88’	E09°59.50’	10	0.4	1
Middle fjord	Tautra	19 September 2018	N63°41.312’	E10°48.001’	20	14.2	32
	Tautra	19 September 2018	N63°41.312’	E10°48.001’	20	14.2	32
		29 August 2019	N63°41.376’	E10°47.934’	10	6.2	14
		23 September 2019	N63°41.376’	E10°47.934’	10	23.5	53
	Ytterøya	16 April 2007	N63°44.98’	E11°06.55’	10	40	
		23 November 2010	N63°44.98’	E11°06.55’	45	1.8	4
		11 November 2014	N63°44.98’	E11°06.55’	50	38.1	86
		30 April 2018	N63°44.98’	E11°06.55’	30	69.90	158
		04 July 2018	N63°44.98’	E11°06.55’	45	44.7	101
		19 June 2019	N63°44.98’	E11°06.55’	20	6.6	15
		15 June 2020	N63°44.98’	E11°06.55’	30	93.8	212
		26 August 2020	N63°44.98’	E11°06.55’	14	15	34
Inner fjord	Beistadfjorden	19 April 2007	N63°56.0198’	E11°05.5610’	10	450	
		25 June 2007	N63°56.0198’	E11°05.5610’	15	600	
		25 October 2007	N63°56.0198’	E11°05.5610’	10	2000	
		02 April 2008	N63°56.0198’	E11°05.5610’	30	9.3	21
		02 April 2008	N63°56.0198’	E11°05.5610’	30	17.7	40
		02 April 2008	N63°56.0198’	E11°05.5610’	20	100	
		03 April 2008	N63°56.0198’	E11°05.5610’	10	1000	
		25 March 2009	N63°56.0198’	E11°05.5610’	15	500	
		23 November 2010	N63°56.0198’	E11°05.5610’	15	1000	
		13 November 2012	N63°56.0198’	E11°05.5610’	10	600	
		05 February 2013	N63°56.0198’	E11°05.5610’	50	9.3	21
		05 February 2013	N63°56.0198’	E11°05.5610’	10	600	
		09 April 2013	N63°56.0198’	E11°05.5610’	10	300	
		12 November 2013	N63°56.0198’	E11°05.5610’	10	1200	
		24 March 2014	N63°56.0198’	E11°05.5610’	10	1000	
		18 June 2014	N63°56.0198’	E11°05.5610’	10	600	
		10 November 2015	N63°56.0198’	E11°05.5610’	20	440	
		29 August 2019	N63°56.0198’	E11°05.5610’	10	62	140
		27 August 2020	N63°56.0198’	E11°05.5610’	5	310.6	702
	Verrasundet	24 October 2006	N63°51.163’	E10°44.006’	40	225.70	510
		25 October 2006	N63°51.163’	E10°44.006’	40	230.1	520
		18 April 2007	N63°51.163’	E10°44.006’	10	750	
		24 October 2007	N63°51.163’	E10°44.006’	25	2000	
		01 April 2008	N63°51.163’	E10°44.006’	40	6000	
		25 March 2009	N63°51.163’	E10°44.006’	10	20	
		28 March 2011	N63°51.163’	E10°44.006’	10	800	
		10 April 2013	N63°51.163’	E10°44.006’	10	100	
		25 March 2014	N63°51.163’	E10°44.006’	20	1500	
		18 June 2014	N63°51.163’	E10°44.006’	10	700	
		11 November 2015	N63°51.163’	E10°44.006’	20	550	
		28 August 2019	N63°51.163’	E10°44.006’	10	110.6	250
		26 August 2020	N63°51.163’	E10°44.006’	10	865.1	1955
	Verrabotten	25 October 2006	N63°49.106’	E10°38.281’	46	79.70	180
		17 April 2007	N63°49.106’	E10°38.281’	51	3000	
		23 October 2007	N63°49.106’	E10°38.281’	20	1240	
		01 April 2008	N63°49.106’	E10°38.281’	41	520	
		28 March 2011	N63°49.106’	E10°38.281’	10	300	
		10 April 2013	N63°49.106’	E10°38.281’	10	200	
		15 November 2013	N63°49.106’	E10°38.281’	10	800	
		26 March 2014	N63°49.106’	E10°38.281’	10	2500	
		18 June 2014	N63°49.106’	E10°38.281’	10	3500	
		12 November 2015	N63°49.106’	E10°38.281’	20	850	
		26 August 2020	N63°49.106’	E10°38.281’	5	471.7	1066
		29 September 2021	N63°49.106’	E10°38.281’	7	497.8	1125

Total *Periphylla periphylla* volume (L^−1^) and abundance (n) are provided.

### Size and weight estimates of *P. periphylla*

During the sampling campaigns from 2018 to 2021, a subset of 30 large (> 10 cm CD) and 20 small (< 10 cm CD) individuals of *P. periphylla* were randomly picked from each catch for size and weight estimations. Sizes of *P. periphylla* were measured using the CD rounded to the nearest centimeter without decimal point and biomass was achieved by weighing individuals on a Marel M1100 scale (> 1 g) right after trawling. Individual developmental stages were assigned based on size according to the classification of Jarms et al. (2002). Stages from 1 to 8 (< 6 mm CD) were considered as embryonic developmental stages, stages 9-14C as “immature” (6–75 mm CD) and stage 14D (> 75 mm CD) as “mature” (Jarms *et al.,* 2002). Individuals < 1 cm (size range between embryonal developmental stage and immature) correspond to an age class of < 1 year (YOY: young-of-the-year). Tissue pieces from the outer umbrella of *P. periphylla* were placed in Ethanol (96%) in microcentrifuge tubes and stored at room temperature until further DNA extraction and population genetic analysis. A power function was fitted to the size–weight relationship. A linear mixed effect model by the lme4 package (Bates *et al.,* 2015) was used to partition size variances between individuals based on sampling location and time (year, month).

### DNA extraction and sequencing

DNA was extracted from in total 190 randomly selected medusae presenting 15–30 specimens per location per sampling day. Small pieces of jelly tissue, preferably some red tissue, were put into 1.5 (2) ml microcentrifuge tubes and left in the fume hood at room temperature overnight to allow the ethanol to evaporate. Cytochrome c oxidase subunit 1 (mtCOI) was selected as a suitable marker. As a basic DNA barcoding region, this mitochondrial marker allows a great deal of information to be gathered and comparisons to be made with many other scyphozoan species for which population genetics data sets exist (Holland *et al.,* 2004; Dawson, 2005; Prieto *et al.,* 2013). DNA extraction was performed by use of (i) modified Chelex rapid-boiling procedure as explained in Granhag et al. (2012), (ii) EZNA mollusk DNA kit and (iii) Qiagen DNeasy kit, following the manufacturer’s instructions. mtCOI amplifications were performed with a variety of primers: HCO2198 and LCO1490 (Folmer *et al.,* 1994), ChryAtlanF1 and ChryAtlanR1 (Abboud *et al.,* 2018), AnthoF1 and AnthoR1 together with QuantaBio and Phire ® Hot Start DNA polymerases and adjusted PCR programs. Due to the generally low success rate, the best working protocol was by the use of Qiagen DNeasy kit, scyphozoan-specific AnthoF1 and AnthoR1 primers with PCR reaction mix of 20 μL and cycling regime of 5 min at 95°C followed by 40 cycles of 94°C (30 s), 54°C (30 s), 72°C (60 s), with a final extension for 7 min at 72°C. PCR products were verified by electrophoresis on an agarose gel (1.5%) in 1xTAE buffer.

PCR products were purified using Illustra GFX PCR DNA and gel band purification kit following the cleaning procedure recommended by the manufacturer. All PCR products were sequenced by commercial service (Eurofins Sequencing Service, Germany, and Macrogen Europe, Netherlands) using the same primer pairs as described above. The resulting nucleotide sequence electropherograms were checked by eye for poor base calls and sequence quality using Chromas Lite 2.1 (Technelysium Pty Ltd). The good-quality sequences were assembled using BioEdit software (Hall, 1999). All publicly available *P. periphylla* sequences from GenBank and Bold (mined 03032021) were combined with our sequences and aligned with the MAFFT online service (Katoh *et al.,* 2019). The sequences were aligned using Q-INS-i strategy, which takes RNA secondary structure into account and gap-opening penalty of 1.53 and gap extension penalty of 0.123. The alignments were visually checked, identical sequences were removed, and poorly aligned regions were excluded prior to the analyses. The alignments are available on request. The sequences reported in this paper have been deposited in the European Molecular Biology Laboratory Nucleotide Sequence Database (GenBank Accession numbers: OY764966-OY764990). Bayesian phylogenetic analyses were performed with MrBayes 3.2.7a (Ronquist *et al.,* 2012). Two independent runs with four Markov chains and 1 600 000 generations were carried out (average standard deviation of split frequencies 0.0094). The model was not chosen prior to the analysis but sampled across the GTR model space with gamma-distributed rate variation across sites and a proportion of invariable sites. The resulting estimates (e.g. tree topology) were posterior probability weighted averages of the models. Maximum likelihood bootstrap support values were calculated from 1000 replicates, using GARLI 2.0.1019 (Zwickl, 2006) with jModelTest 0.1.1 (Posada, 2008) AICc criterion selected model (TIM2 + I + G).

### Population genetic analysis

Estimates of genetic variation were obtained for all the samples as well as for samples grouped by different seasons and geographical areas. The main genetic structure and differentiation analysis were calculated, for example, the nucleotide diversity (p) and haplotype diversity (h) were estimated using the program DnaSP v5 (Librado & Rozas, 2009) and genetic differentiation was calculated by means of pairwise FST values using 10 000 permutations in ARLEQUIN 3.1 (Excoffier *et al.,* 1992) within the analysis of molecular variance (AMOVA) framework (Excoffier *et al.,* 1992). A median-joining network showing the relationships between the mtDNA haplotypes was constructed using the PopART (http://popart.otago.ac.nz/howtocite.shtml;Bandelt *et al.,* 2000). Tests for population expansion based on Tajima’s D and Fu’s FS (Ramos-Onsins and Rozas, 2002) were carried out using DnaSP v5. Significance levels were corrected using Bonferroni correction.

## RESULTS

### *Periphylla periphylla* population size

**Fig. 2:**
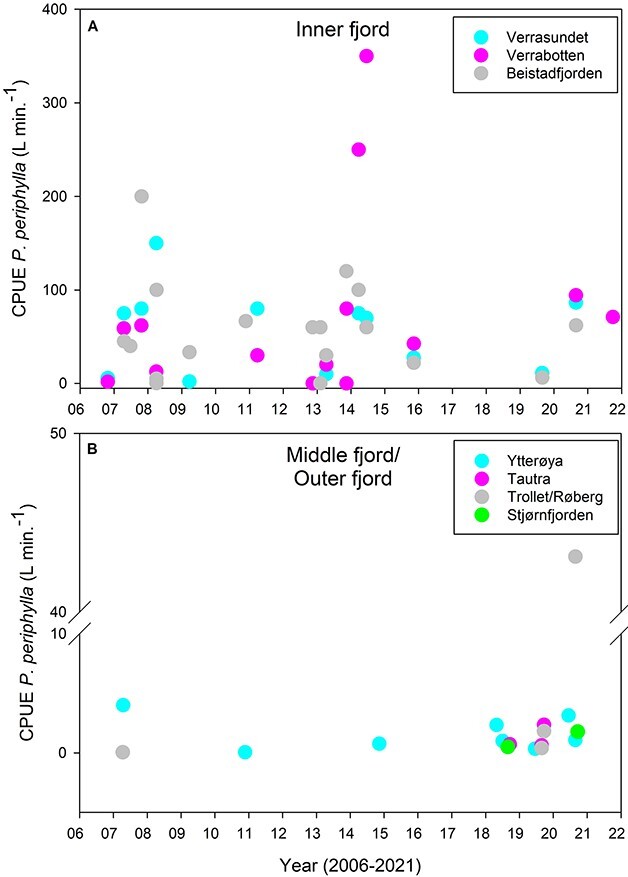
*P. periphylla* catches (L min.^−1^) calculated as CPUE during bottom trawl activities in Trondheimsfjorden in the period 2006–2021 in the Inner fjord (stations Verrasundet, Verrabotten and Beistadfjorden) (**A**) and Middle and Outer fjord (Ytterøya, Tautra, Trollet/Røberg and Stjørnfjorden) (**B**).

Overall, *P. periphylla* was caught at all stations within Trondheimsfjorden. Abundance of *P. periphylla* in the inner part of the fjord was higher compared with the middle and outer part of the fjord. The highest CPUE of 350 *P. periphylla* (L min^−1^) was estimated in June 2014 in the inner fjord (Verrabotten, Fig. 2A). In general, no temporal trend of biomass increases or decreases of *P. periphylla* was observed in the inner part of Trondheimsfjorden (Fig. 2A). The amount of *P. periphylla* caught in the middle and outer fjord (Fig. 2B) was 100 times lower compared with the inner fjord. No differences in biomass were observed between stations in the outer and middle fjord and no clear temporal trend was observed over the last 15 years.

### Size and age distribution

**Fig. 3:**
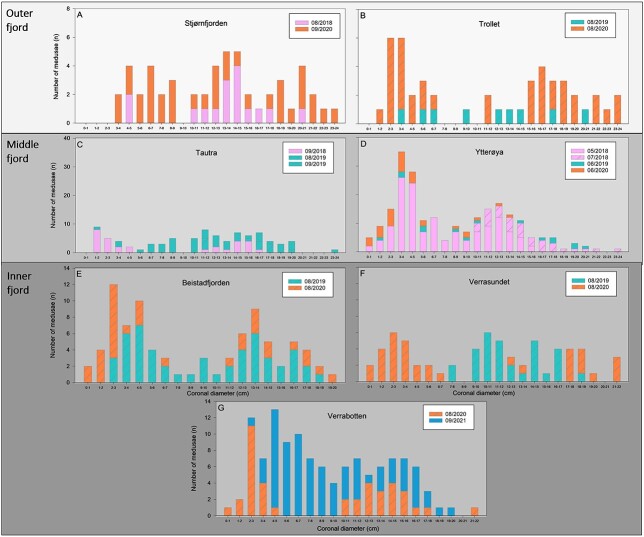
Size distribution of *P. periphylla* medusae caught in Trondheimsfjorden during bottom trawling activities at the Outer fjord stations Stjørnfjorden (**A**) and Trollet (**B**), the Middle fjord stations Tautra (**C**) and Ytterøya (**D**), and the Inner fjord stations Beistadfjorden (**E**), Verrasundet (**F**) and Verrabotten (**G**) during the period of spring to autumn (2018–2021).

Specimens collected during cruises from 2018 to 2021 in Trondheimsfjorden varied in size between locations and years, with an average size in the category of mature specimen of 9.0 cm ± 5.1 in 2018 (*n* = 248), 12.1 cm ± 5.3 in 2019 (*n* = 222) and 9.13 cm ± 6.9 in 2020 (*n* = 293) (Fig. 3). The outer fjord stations were characterised by a complete lack of individuals < 1 cm corresponding to an age class of < 1 year (young-of-the-year, YOY). Some immature *P. periphylla* (< 7.5 cm) were found at the outer fjord stations, especially in 2020, while the majority of specimens where mature medusae > 7.5 cm with individuals up to 24 cm CD. Overall, very large *P. periphylla* (CD > 22 cm) were only found at the middle and outer fjord stations (Fig. 3D–G). The two stations sampled in the middle fjord showed differences in size distribution. While at Tautra, specimens < 1 cm (< 1 year of age) were missing (Fig. 3C), some YOY medusae were found at Ytterøya in 2018 (May) and 2020 (June) (Fig. 3D). While a relative even distribution of size classes from 1 to 20 cm was observed at Tautra, the majority of medusae found at Ytterøya belonged to the embryonal developmental stage or were immature < 7.5 cm (ca. 1–4 years of age). This was particularly true for 2018 (May) and 2020 (June). In 2018, mature specimens (> 7.5 cm) occurred with a majority in the size categories 8–15 cm CD and up to a maximum of 24 cm CD. In the inner fjord, YOY *P. periphylla* (< 1 cm) were found at all three stations (Fig. 3E–G) but only in 2020 and at low numbers. Overall, the majority of individuals were in the immature/embryonal developmental stage in 2020 with a CD < 7 cm or mature specimens with a CD > 10 cm at all three stations. The intermediate size classes from 7 to 11 cm were entirely missing in 2020 while they were present in 2019 (Beistadfjorden, Verrasundet) and 2021 (Verrabotten). The maximum size of *P. periphylla* specimens in the inner fjord was 20 (Beistadfjorden) and 22 cm (Verrasundet, Verrabotten).

### Size–weight relationship

**Fig. 4:**
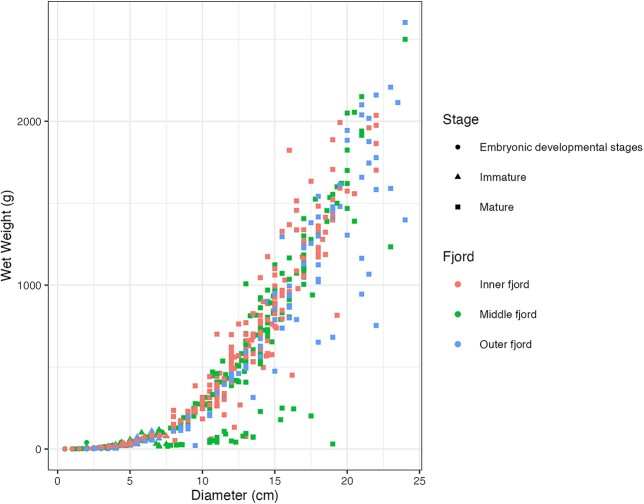
Coronal diameter (CD in cm) to wet weight (g) relationship of *P. periphylla* medusae sampled during bottom trawling activities in three different areas of Trondheimsfjorden: Inner fjord (left panel), Middle fjord (intermediate panel) and Outer fjord (right panel) in the years 2018–2021. According to size, specimens were categorised as “embryonic developmental stage” (CD < 6 mm; dots), “immature” (CD: 6 to 57.3 ± 17.8 mm; triangles) and “mature” (>75.1 mm; squares) following data provided by Jarms et al. (2002).

**Fig. 5:**
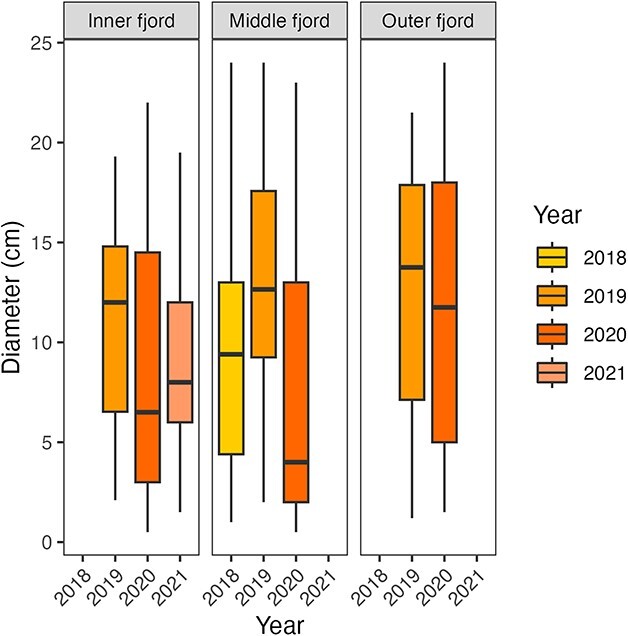
Coronal diameter (CD in cm) of *P. periphylla* medusae sampled during bottom trawling activities in three different areas of Trondheimsfjorden (Inner fjord, Middle fjord and Outer fjord) in the years 2018–2021.

The CD and the wet weight of the *P. periphylla* medusae sampled in three different areas of Trondheimsfjorden showed an exponential relationship described by the function *y* = 0.31 *x*^2.92^ for individuals in the inner fjord (red color-coded individuals; Fig. 4) and *y* = 0.58 *x*^2.56^ for individuals sampled in the outer and middle fjord (blue and green color-coded individuals; Fig. 4). For both functions, between 89 and 91% of the total variation in weight could be explained by its relation to diameter. Partitioning the data further into fjord location and year did not indicate any population size trend (either decreasing or increasing) over the years (Fig. 4). To investigate the effect of location on the size of *P. periphylla*, we fitted a random intercept model of scaled and centered *P. periphylla* diameters with crossed random effects of year and month, using the lmer function from the lme4 package. Our results revealed that 23% of the variance in the data can be explained by the random effect month meanwhile year had negligible effect (< 0.05%; Fig. 5). However, what causes the majority (77%) of the variance is unknown. Our results showed the presence of smaller individuals (β = −0.23 +/− 0.28) in the inner part of the fjord meanwhile the middle and the outer fjord showed the presence of bigger individuals (β = 0.35 +/− 0.14 for the middle fjord and β = 0.32 +/− 0.12 for the outer fjord). These findings confirm our H2 hypothesis indicating that the inner part of the fjord harbors smaller individuals indicating local reproduction.

### Genetic structure and differentiation

**Fig. 6:**
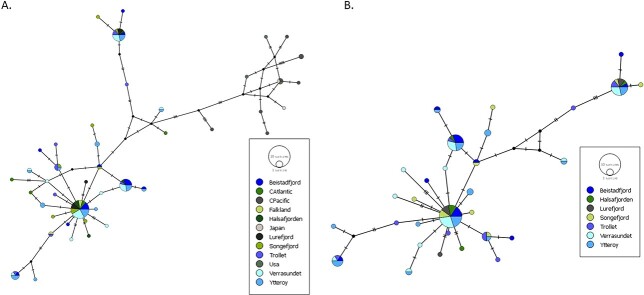
(**A**) Median-joining network within *P. periphylla* showing the relationships between the 42 haplotypes found globally detected by sequencing the mitochondrial DNA cytochrome oxidase I (COI) region and (**B**) median-joining network within *P. periphylla* showing the relationships between the 30 haplotypes found in Norway detected by sequencing the mitochondrial DNA COI region. Circle sizes are approximately proportional to haplotype frequency: the smallest circle represents a single individual; the largest circle represents 25 individuals. Each connection represents a single mutation and small open black dots represent missing intermediate haplotypes.

In general, the success rate of COI sequencing was low. The 190 *P. periphylla* individuals used for molecular analyses resulted in 93 *P. periphylla* good-quality sequences for the COI region (alignment of 552 bp). On a global scale, 114 specimens with 42 haplotypes defined by 52 segregating sites, of which 32 were parsimony informative, were recorded (Fig. 6A). In Norway, 30 haplotypes defined by 37 segregating sites, of which 20 were parsimony informative, were recorded (Fig. 6B). The haplotype network showed that the most frequently found haplotype occurred at various locations globally and in Norway. Verrasundet, the inner part of Trondheimsfjorden, shared the least haplotypes with other areas. Singletons from the same geographic regions did not cluster in monophyletic groups, while some haplotypes from the Pacific side of Canada and US regions did. In Norway, haplotype richness was high (*h* = 0.884 ± 0.00046), but differentiation among haplotypes was modest (*π* = 0.00771, Table II). The highest haplotype richness was calculated in Trollet, Trondheimsfjorden (0.96) and Sognefjorden (0.93) and lowest in Lurefjorden (0.73) whereas the highest COI nucleotide diversity was calculated in Beistadfjorden, Trondheimsfjorden (1%) and the lowest in Lurefjorden (0.68%). The pairwise FST values here indicated significant population differentiation between Trollet and Verrasundet, Ytterøya and Verrasundet in Trondheimsfjorden, and Lurefjorden and Sognefjorden, but after Bonferoni correction, no significant population differentiation was detected (Table III).

**Table II:** Sample sizes and standard diversity indices for COI sequences of P. periphylla medusae sampled in seven different areas in Norway

Area	Population	*N*	Nh	H ± sd	*π*
Trondheimsfjord	Beistadfjord	15	9	0.89 ± 0.06	0.99
¨	Trollet	10	8	0.96 ± 0.06	0.82
¨	Ytterøy	24	12	0.92 ± 0.03	0.73
¨	Verrasundet	22	10	0.87 ± 0.06	0.7
Halsafjord	Halsafjord	6	4	0.80 ± 0.17	0.89
Lurefjord	Lurefjord	6	3	0.73 ± 0.16	0.68
Sognefjord	Sognefjord	10	8	0.93 ± 0.08	0.99

Seventy-eight sequences from this study and 15 sequences obtained from Bold were analysed. The sample size (*N*), the number of haplotypes per location (Nh), haplotype diversity (H) and nucleotide diversity (also called heterozygosity, π) are shown.

**Table III:** Pairwise FST values between geographic regions based on 10 000 permutations

Population	Beistadfjorden	Trollet	Ytterøy	Verrasundet	Sognefjorden	Lurefjorden	Halsafjorden
Beistadfjorden							
Trollet	0.05						
Ytterøy	0.05	0.07					
Verrasundet	−0.01	0.12^*^	0.13^*^				
Sognefjorden	0.04	0.00	0.09^*^	0.04			
Lurefjorden	0.06	0.04	0.20^*^	0.03	−0.08		
Halsafjorden	−0.02	−0.03	0.03	−0.01	−0.10	−0.10	

^*^ indicates significant differentiation uncorrected *P* < 0.05.

## DISCUSSION

### Long-term trends in *P. periphylla* population size

Reliable predictions on jellyfish bloom intensities and frequencies are limited by a lack of knowledge related to their population and life-history dynamics. This is mainly due to a lack of consistent data on jellyfish population size, reproduction rates, mortality and spatiotemporal distributions. Moreover, intra- and inter-annual variations are usually based on snapshot surveys at a low spatiotemporal resolution or are completely missing. In the last decades, progress has been made, especially thanks to recent efforts to collect time series data and citizen science observations (Condon *et al.,* 2013).

In the present study, no significant increase in *P. periphylla* population size in Trondheimsfjorden was found over the 15 years examined. The CPUE remained at similar levels throughout the period 2006–2021 and no clear trends could be detected thereby rejecting our first hypothesis (H1). This is in contrast to previous studies that reported on a strong decline in CPUE for cod in Trondheimsfjorden during the period 2007–2014, followed by an increase in CPUE for *P. periphylla* (Tiller *et al.,* 2015)*.* The possibility of mass occurrences of *P. periphylla* in Trondheimsfjorden raised major concerns as it could potentially lead to a regime shift from a fish- to a jellyfish-dominated ecosystem (Tiller *et al.,* 2014, 2015). These studies focused on the socioeconomic consequences related to the dominance of *P. periphylla* including declines in fish stocks, challenges for ecosystem management and negative effects on local fisheries, specifically in the innermost parts of the fjord. Based on our CPUE data, strong variation between sampling years and locations in Trondheimsfjorden occurred during the last 15 years with 100-fold higher CPUE obtained in the innermost part of Trondheimsfjorden compared with middle and outer fjord regions. Although this dataset is based on snap-shot sampling and doesn’t tackle entirely patchy and seasonal bloom distribution patterns, some general trends, i.e. on biomass accumulation and life-history dynamics of *P. periphylla* populations specifically in the innermost parts of the fjord were obtained. The causes and consequences of these findings are discussed in detail in the context of water-mass exchange, fjord topography, advection/retention times and recruitment success in the following discussion sections.

### Recruitment and reproduction success of *P. Periphylla* in Trondheimsfjorden

The helmet jellyfish *P. periphylla* is characterised by a holopelagic life cycle reproducing sexually and the development from egg to medusa happens directly without including the intermediate planula, polyp and ephyra stages (Jarms *et al.,* 1999, 2002). Little is known about its reproduction rate and lifespan, although a high longevity of this species has previously been assumed (Jarms *et al.,* 1999). Overall, *P. periphylla* individuals in Trondheimsfjorden showed a wide size distribution with large specimens (CD > 20 cm) found in all three regions and YOY specimens (< 1 cm) found in the middle and inner part of the fjord. Maximum sizes of *P. periphylla* individuals in Trondheimsfjorden can be considered as large compared with other Norwegian fjords (Bamstedt *et al.,* 2020) and other Atlantic regions (Lucas and Reed, 2010). Since fecundity can be related to female size to some degree (Bamstedt, 2022), the occurrence of large specimens (> 20 cm CD) might provide indication for local reproduction of *P. periphylla* in Trondheimsfjorden. Young recruits (< 7.5 cm CD) were well represented in all three fjord regions, often even dominating the population. This further supports the assumption of local reproduction and self-sustaining population of *P. periphylla* in Trondheimsfjorden thus confirming our second hypothesis (H2).

Overall, the size–weight relationship of *P. periphylla* could be best described by a power function with an exponent of 2.68. This is similar to the equation provided earlier on the size–weight relationship of *P. periphylla* with an exponent of 2.98 (Bamstedt, 2022). However, size–weight relationships of populations in the middle and outer fjord area deviated significantly from the ones in the inner fjord showing low weights relative to CD size. The size–weight relationships found for inner fjord populations could best be described by a power function with an exponent of 2.92. The findings in this study were in accordance with the relationship previously identified for *P. periphylla* in Norwegian fjords (Bamstedt, 2022). Between- and within-fjord variations in the size–weight relationship of populations could result from changes in biotic and abiotic conditions that can affect *P. periphylla* condition and growth. Such alterations in environmental conditions are, for example, considered to induce mass decay in *P. periphylla* biomass (Bozman *et al.,* 2018) or parasitic infestation (Solheim, 2012).

### The inner fjord: a hotspot of *P. periphylla* reproduction?

Biomass accumulation and retention times of *P. periphylla* in fjords are considered to be directly related to basin topography, sill depth, water exchange rates, light attenuation and vertical migration behavior (Youngbluth and Bamstedt, 2001; Sornes *et al.,* 2007). Trondheimsfjorden is considered a *P. periphylla* hotspot with high population size reported from 2002 onwards, especially in the innermost fjord regions (Tiller *et al.,* 2017). Due to a strong accumulation/retention and broad size distribution ranges, the innermost fjord is considered to harbor it’s “mother population” and most recruitment is assumed to happen there (Solheim, 2012). In our study, a size-dependency of *P. periphylla* in the outer, middle and inner fjord was detected showing a higher proportion of small-sized individuals in the inner fjord. YOY size classes of *P. periphylla* (< 1 cm, stage 9–11, < 1 year of age) were sampled mainly at the inner fjord stations Verrasundet, Verrasundet and Beistadfjorden and one innermost station in the middle fjord (Ytterøya). The presence of very small specimens (<1 cm) points at a local reproduction of *P. periphylla* in the inner part of Trondheimsfjorden where populations accumulate, thus confirming hypothesis 2.

Overall, a wide range of *P. periphylla* size classes with a total size spectrum of medusae ranging from 0 to 24 cm CD could be sampled in Trondheimsfjorden during the period 2018–2021. Despite the fact that the current dataset has its constraints regarding spatiotemporal resolution, it provides snapshots of *P. periphylla* occurrence, population structure and connectivity from multiple sampling years and fjord locations. The fact that a large proportion of small specimens (size class < 7 cm) was documented in our study (in various years and locations) provides indication for recruitment success of *P. periphylla* in Trondheimsfjorden. This is in contrast to i.e. Lurefjorden where a 3-year recruitment failure was documented by the lack of young recruits (Bamstedt, 2022). Short intervals of deep-water renewal of fjord basins are considered a possible cause for recruitment failure in *P. periphylla* (Bamstedt, 2022) due to the fact that eggs and embryonal developmental stages thrive in the deep layers of the fjords. Thus, short intervals of deep-water replacement can lead to a continuous flushing out of young recruits. In Trondheimsfjorden, the intervals of deep-water renewal are subject to seasonal inflow and replacement of bottom water in all three deep basins each spring (inner, middle and outer fjord basins) (Jacobson, 1983). However, the replacement of bottom water in the inner fjord usually lags behind due to the small cross-section and shallow sill at Skarnsund that function as a barrier for water exchange (Jacobson, 1983). The fact that most young recruits (<1 cm, < 1 year) of *P. periphylla* were found at Beistadfjorden, Verrasundet and Verrabotten in 2020 could point at a reduced deep-water renewal in the inner fjord in this specific year. Further, the overall absence of YOY in the outer fjord regions (incl. Tautra) could be the result of short deep-water renewal intervals that are known to affect local recruitment (Bamstedt, 2022). This could result in a continuous flushing of eggs and young recruits from the outer deep basins and an enhanced dispersal of *P. periphylla* out of Trondheimsfjorden and into adjacent waters resulting in a northward advection via the Norwegian Coastal Current (Tiller *et al.,* 2017).

Despite strong indication for a continuous reproduction in this coronate species throughout the year (Tiemann and Jarms, 2010; Bamstedt *et al.,* 2020), we found that part of the variation (31%) in CD size could be attributed to the sampling month and deviations in the size–weight relationship for specific age groups. This could point to some degree of seasonality in *P. periphylla* reproduction in Trondheimsfjorden, similar to what has been observed in Vefsnfjorden in Northern Norway (Bozman *et al.,* 2018). Mortality might also change with season in relation to environmental drivers that can affect *P. periphylla* growth, survival and performance in specific years and seasons when e.g. mass mortalities (Bozman *et al.,* 2018), parasitic infestation (Solheim, 2012) and nutritional constraints (Sornes *et al.,* 2008) occur. So far, the role of predation by other vertebrate and invertebrate species on *P. periphylla* and their impact on population regulation are understudied. However, there is indication that intense predation events e.g. by anemones happen, as have been observed in Lurefjorden and Sognefjorden (Jarms and Tiemann, 2004) as well as in Trondheimsfjorden (Jarnegren, pers. comm.).

### Population structure and connectivity of *P. periphylla* in Trondheimsfjorden and adjacent waters

The most consistent result of the COI analyses of *P. periphylla* is that no clear population structure was detected among the different areas inside Trondheimsfjorden or between Trondheimsfjorden and the more oceanic areas outside the fjord. This finding suggests the presence of mixing populations and is in agreement with H3 of our initial hypotheses. Despite a generally low success rate in sequencing and use of single mitochondrial marker, such a low geographic population structure might be due to strong dispersal and frequent deep-water renewal of the fjord basins that keep populations well-mixed and suggest a high gene flow.

The ecological and biological traits of *P. periphylla,* such as opportunistic feeding on almost all zooplankton groups, an extended breeding period with solely holoplanktonic life cycle (Fossa, 1992; Jarms *et al.,* 1999; Youngbluth and Bamstedt, 2001) and survival in a wide range of environmental conditions are all factors known to support dispersal and admixture. Similar results have been found for other jellyfish species sharing the same traits, such as *Pelagia noctiluca* (Stopar *et al.,* 2010). In contrast, more geographically structured intraspecific phylogenies have been detected from jellyfish taxa that show a metagenetic life cycle including a sessile polyp phase (Schroth *et al.,* 2002).

Advective water-mass exchange between the fjord and the open sea has been speculated to be a crucial factor with a strong potential to affect fjord populations. For example, shallow sills at the mouths of fjords have been suggested to restrict gene flow of the mesopelagic fish *Benthosema glaciale* commonly found in the Norwegian Sea and several west Norwegian fjords (Suneetha and Salvanes, 2001; Kristoffersen and Salvanes, 2009). For *P. periphylla*, the study by Sørnes et al. (Sornes *et al.,* 2007) presented a model to explain *P. periphylla* retention in three Norwegian fjords. This model was based on vertical distribution, advection and light attenuation as a governing factor for the vertical distribution. Although the model gives a logical explanation of why the three fjords can have a sustainable population of *P. periphylla*, it does not explain the mechanism behind the strong population differences in abundance and size distribution between the studied fjords (Sornes *et al.,* 2007). In Trondheimsfjorden, the small genetic diversity and high proportion of small specimens detected in the inner fjord point at a true bloom resulting from local reproduction (Graham *et al.,* 2001), whereas more mixed areas in the outer and middle fjord (e.g. Stjørnfjorden, Trollet, Tautra) promote a mixture of true and apparent aggregations, resulting from the combination of physical advection/retention and local reproduction. This could explain population variations between different areas in the fjords. However, it is important to keep in mind that lack of population structure does not necessarily imply demographic connectivity across the areas (Lowe and Allendorf, 2010; Drake *et al.,* 2022). Hence, more detailed analysis would be needed.

The present study provides new insights into the fine scale population structure and origin of jellyfish blooms within one fjord as well as between different fjord ecosystems. By using a combination of field observation, modeling and molecular tools, the study contributes to a better understanding on the factors that trigger jellyfish bloom formation, population structure and connectivity in fjord ecosystems and adjacent waters, thus enhancing our knowledge on jellyfish bloom dynamics in marine ecosystems.

## CONCLUSION

Fjord ecosystems provide unique habitats that are of high ecological and economical relevance and, at the same time, are prone to a variety of anthropogenic stressors (i.e. climate change, pollution, overfishing). Using a combination of approaches including time-series trawl data, sampling at high spatio-temporal resolution during recent years and population genetics provided insights into the dynamics, structure and connectivity of the helmet jellyfish (*P. periphylla*) in Trondheimsfjorden and adjacent waters. Such knowledge on ecosystem dynamics and environmental status of fjords over longer timescales are essential for the development of reliable and sustainable ecosystem management strategies thus advancing management approaches that meet ecosystems’ and stakeholders’ demands.

## Data Availability

The sequences reported in the study have been deposited in the European Nucleotide Archive repository with accession numbers: OY764966-OY764990.
